# Microbiota Depletion Promotes Human Rotavirus Replication in an Adult Mouse Model

**DOI:** 10.3390/biomedicines9070846

**Published:** 2021-07-20

**Authors:** Roberto Gozalbo-Rovira, Cristina Santiso-Bellón, Javier Buesa, Antonio Rubio-del-Campo, Susana Vila-Vicent, Carlos Muñoz, María J. Yebra, Vicente Monedero, Jesús Rodríguez-Díaz

**Affiliations:** 1 Department of Microbiology, School of Medicine, University of Valencia, Av. Blasco Ibáñez 17, 46010 Valencia, Spain; rovigoro@uv.es (R.G.-R.); cristina.santiso@uv.es (C.S.-B.); javier.buesa@uv.es (J.B.); susana.vila@uv.es (S.V.-V.); carlos.munoz@uv.es (C.M.); 2 Hospital Clínico Universitario de Valencia, Instituto de Investigación INCLIVA, 46010 Valencia, Spain; 3 Department of Biotechnology, IATA-CSIC, Av. Agustín Escardino 7, Paterna, 46980 Valencia, Spain; anrucam@iata.csic.es (A.R.-d.-C.); yebra@iata.csic.es (M.J.Y.)

**Keywords:** rotavirus, antibiotic, microbiota, mice, virus shedding

## Abstract

Intestinal microbiota-virus-host interaction has emerged as a key factor in mediating enteric virus pathogenicity. With the aim of analyzing whether human gut bacteria improve the inefficient replication of human rotavirus in mice, we performed fecal microbiota transplant (FMT) with healthy infants as donors in antibiotic-treated mice. We showed that a simple antibiotic treatment, irrespective of FMT, resulted in viral shedding for 6 days after challenge with the human rotavirus G1P[8] genotype Wa strain (RVwa). Rotavirus titers in feces were also significantly higher in antibiotic-treated animals with or without FMT but they were decreased in animals subject to self-FMT, where a partial re-establishment of specific bacterial taxons was evidenced. Microbial composition analysis revealed profound changes in the intestinal microbiota of antibiotic-treated animals, whereas some bacterial groups, including members of *Lactobacillus*, *Bilophila*, *Mucispirillum,* and *Oscillospira*, recovered after self-FMT. In antibiotic-treated and FMT animals where the virus replicated more efficiently, differences were observed in gene expression of immune mediators, such as IL1β and CXCL15, as well as in the fucosyltransferase FUT2, responsible for H-type antigen synthesis in the small intestine. Collectively, our results suggest that antibiotic-induced microbiota depletion eradicates the microbial taxa that restrict human rotavirus infectivity in mice.

## 1. Introduction

Diarrheal disease is the second leading cause of death worldwide in children under five years of age, accounting for around 525,000 deaths each year. Rotavirus (RV) is among the predominant causes of non-bacterial acute gastroenteritis in infant and young children, with an estimated 150,000 deaths per year, mostly in developing countries [1]. The gut is a very complex ecosystem, with multiple interactions between the host immune system, glycobiology, resident microbiota, and viruses responsible for gastroenteritis [2,3,4,5]. A link between human RV infection and intestinal bacterial populations has been revealed from analysis of the microbiota in population groups displaying different vaccine take after inoculation with RV live vaccines [6,7]. However, the role of commensal microbiota in RV infectivity is still controversial. Early evidences pointed towards a positive effect, showing reduced RV infectivity in germ-free or antibiotic-treated mice [8]. Contrarily, some microorganisms, including probiotic bacteria, directly interact with RV and potentially block their binding to epithelial receptors, counteracting infection [9].

RV attach to glycans of the histo-blood group antigen (HBGA) expressed in the gastrointestinal tract. In humans, fucosylation of O-linked HBGA at this location is controlled by *FUT2* and *FUT3* gene expression, and it has been demonstrated that the glycosylation patterns of those receptors could be altered by the action of specific commensal bacteria [10]. The main human RV genotype (G1P[8]) recognizes fucosylated HBGAs, such as H-type 1 [11], synthesized by FUT2 activity, and Lewis b antigens [12], synthesized by FUT3, but these viruses show inefficient spread or no replication at all in the mouse model [13].

With the aim of obtaining a versatile animal model for human RV infection, we tested whether engraftment of infant intestinal microbiota would permit G1P[8] RV infection in the mouse model. We showed that a simple microbial ablation through antibiotics was sufficient to promote human RV infection in mice, highlighting a role for intestinal microbiota in suppressing these RVs in the murine model.

## 2. Materials and Methods

### 2.1. Rotavirus Stock Preparation

A human RVwa strain was produced as previously described [14] with modifications. Briefly, 10 MA104-confluent 150 cm^2^ flasks (approximately 1.5 × 10^7^ cells/flask) were infected with Wa RV at a multiplicity of infection (MOI) of 0.1 and the flasks were kept at 37 °C for one week. After infection the virus was pelleted at 100,000× *g* for 2 h in a Himac R25ST-0507 rotor coupled to a Himac CR-30N× centrifuge. The pelleted virus was resuspended in TNC (20 mM Tris-HCl pH 8.0, 100 mM NaCl, 1 mM CaCl_2_) and ultra-centrifuged in a sucrose gradient (30–70%) in TNC. The gradient was run in a SW41 rotor coupled to a Beckman L80 ultracentrifuge at 150,000× *g* for two hours. The band containing RV was collected and the virus was finally recovered by pelleting in the SW41 rotor coupled to the Beckman L80 ultracentrifuge for 2 h at 150,000× *g* and resuspended in TNC. After preparation the RV Wa stock was titrated by qPCR, as previously described [15].

### 2.2. Donor Microbiota Preparation

Stool samples from four healthy human infants between one and three months of age were collected and resuspended in a solution consisting of 80%×-concentrated brain–heart infusion (Pronadisa, Madrid, Spain), supplemented with 0.1% cysteine, and 20% of a 200 g/L skim milk (Scharlab, Barcelona, Spain) solution. This mixture was then diluted 1:2 (*v*/*v*) in the same medium and stored in aliquots at −80 °C. Mice fecal pellets from the animal groups were collected before the experiments and preserved using the same procedure.

### 2.3. Antibiotic Treatment and Microbiota Stool Transplant

Four groups of five C57BL/6J female 6 week-old mice were used in the present study. Three groups were treated with an antibiotic cocktail composed of 1 g/L ampicillin, 1 g/L metronidazole, 1 g/L neomycin, and 0.5 g/L vancomycin in drinking water, as previous described with modifications [16]. To diminish intestinal microbiota load, the animals were given the antibiotic-containing water ad libitum for three weeks and the antibiotic cocktail was renewed every three days. To determine the number of viable bacteria in mice stools, samples were collected at the beginning of the experiment and the day before RV infection, and serially diluted in brain heart infusion supplemented with 0.1% cysteine. The dilutions were plated in Wilkins–Chalgren medium containing 1.5% agar and incubated in anaerobic conditions (AnaeroGen, Sigma, Madrid, Spain) at 37 °C for 48 h. Mice were fed a standard diet until one week before fecal transplantation, when it was substituted by purified-defined germ-free diet (AIN-93G, Envigo, Barcelona, Spain ).

The control group was maintained without antibiotics. Twenty-four hours after antibiotic treatment completion, two groups of mice were subjected to fecal material transplantation (FMT), as previously described with modifications [17]. One group of mice was transplanted with the preserved microbiota from the same group of mice before antibiotic treatment (100 μL of prepared fecal material per mice for three consecutive days through oral gavage). A second group of mice received a FMT using the same procedure with a pool of bacteria from infant feces (100 μL of a pooled mix of four healthy infants for three days through oral gavage).

### 2.4. Rotavirus Challenge

Six days after FMT the mice were orally inoculated with 1 × 10^10^ genome copy equivalent (GCE) of RVwa in 100 μL of TNC. After RV dosing, the antibiotic-treated group without FMT continued the drinking water antibiotic treatment, whereas in the FMT mice groups, antibiotics were omitted from water from the day before microbiota transplant. Stool samples were collected daily for 7 days and kept at −20 °C. The mice were euthanized at 7 days post-infection (dpi) and the small intestine was removed and stored in RNAlater (Sigma) at −80 °C for further analysis.

### 2.5. Quantification of Rotavirus from Stool Samples by RT-qPCR

We extracted RNA from mice stool samples using the NucleoSpin RNA Virus (Macherey-Nagel, Duren, Germany) kit following the manufacturer’s instructions. The primers and probe sequences utilized for RT-qPCR have previously been described [15]. Amplification of RNA samples was performed with one-step TaqMan RT-qPCR using the RNA UltraSense One-Step quantitative system (Thermo Fisher Scientific, Madrid, Spain). The standard curve was generated by serial end-point dilution, amplifying 10-fold dilutions of the quantified plasmid containing the RV target sequence by RT-qPCR in triplicates.

### 2.6. Quantification of Cytokine and Glycosyltransferase mRNA Expression Level

Then, 100 mg of tissue from the small intestine of infected mice were homogenized in 1 mL of Trizol (Thermo Fisher Scientific) using a Polytron PT10-35 GT (Thermo Fisher Scientific) at 16,000 rpm. After tissue disruption the RNA was purified following the manufacturer’s instructions and the RNA finally resuspended in 50 μL of DEPC-treated water. The RNA was treated with RNAse-free DNAse I (Thermo Fisher Scientific) to remove the contaminant DNA and retro-transcribed to cDNA using the SuperScript III enzyme (Thermo Fisher Scientific) and random primers following the manufacturer’s instructions. The cDNA obtained was kept at −20 °C until use. Expression levels of genes encoding cytokines *IL1β, IL4, IL6, CXCL15, IL10, IL12, IL13, TNFα, IFNγ,* and *TLR2* were studied in a LightCycler480 Instrument SW1.5 (Roche Life Science, Basel, Switzerland) and the expression analysis performed with the Rest Software [18]. The expression level of *GAPDH* and *RPLPO* housekeeping genes was used as reference. A list of primers employed can be found in Supplementary Table S1.

Glycosyltransferase *FUT2* gene expression was also analyzed by RT-qPCR. Primer and probe preparation for the gene was purchased from Integrated DNA Technologies, Inc. (IDT, assay ID: Mm.PT.58.50508299). The NZYSpeedy qPCR Probe Master Mix (NZY, Lisbon, Portugal) was utilized. The qPCR was run in the StepOnePlus Real-Time PCR System (Applied Biosystems) and expression analysis was performed with Rest Software [18], using HPRT gene expression (assay ID: Mm.PT.39a.22214828) as a reference.

### 2.7. Microbiota Profiling in Mice Stool Samples

Total rDNA 16S in mice feces was quantified by qPCR as previously described [19]. To assess microbiota composition, DNA was extracted from mice fecal pellets obtained prior to RVwa inoculation from all experimental groups with the Master Pure™ kit (Epicentre), including a bead beater treatment with 0.5 g of 0.1 mm glass beads in the lysis step (FastPrep 24-5G Homogenizer; MP Biomedicals, CA, USA), followed by final purification of the extracted DNA samples with mi-PCR Purification kit (Metabion, Planegg-Steinkirchen, Germany). DNA was quantified with a Qubit 2.0 fluorometer (Invitrogen, Thermo Fisher Scientific, Madrid, Spain). Bar-coded amplicons of the 16S rDNA V3–V4 region, multiplexed using Nextera XT Index Kit, were subject to 2 × 300 bp paired-end run in a MiSeq-Illumina platform (SCSIE, University of Valencia, Valencia, Spain). Data were demultiplexed using Illumina bcl2fastq© program and reads were checked for quality, adapter trimmed and filtered using AfterQC and FastQC v0.11.8 (http://www.bioinformatics.babraham.ac.uk, accessed 10 May 2021) tools. QIIME software V1.9.1 [20] was used to analyze MiSeq sequencing data, including forward and reverse reads joining, chimera removal, data filtering, and taxonomic annotation. Chimeric sequences were removed from the reads using the USEARCH 6.1 algorithm. Reads were clustered into operational taxonomic units (OTUs) based on a 97% identity threshold value. Sequence were aligned to the Greengenes core reference database (version 13.8) using PyNAST. Taxonomic assignment was made using the UCLUST classifier. A total of 4,356,240 non-chimeric reads were obtained, with a mean of 207,440 sequences per sample. Datasets were rarefied to the minimum library size (43,791 reads) and normalized by total-sum scaling prior analysis with Calypso 8.84 (http://cgenome.net/calypso/ accessed 10 May 2021) and MicrobiomeAnalyst (https://www.microbiomeanalyst.ca/ accessed 10 May 2021; [21] software pipelines.

## 3. Results

### 3.1. Mice with Depleted Microbiota and Transplanted with Human Feces Shed Wa Rotavirus for 6 Days

**Figure 1:**
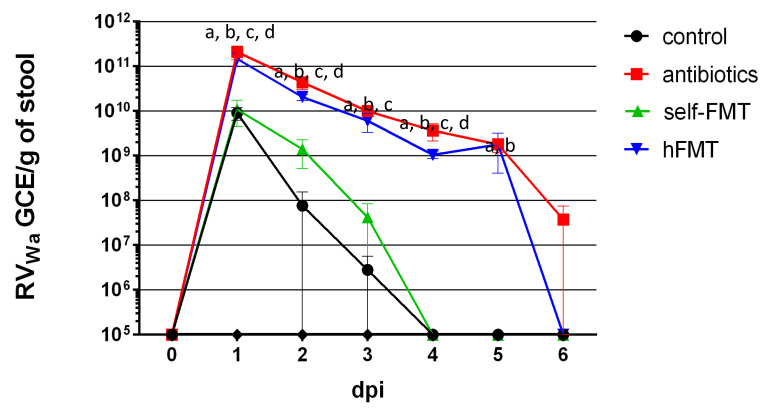
Rotavirus Wa shedding in mice stool. GCE/g of stool are presented for control mice, Ab-treated mice and mice subject to hFMT and self-FMT, respectively. The mice were followed for 6 days and euthanized on day 7. The letters indicate statistically significant differences (*p <* 0.05) in the GCE between the groups (a, Ab vs. control; b, Ab vs. self-FMT; c, hFMT vs. control; d, hFMT vs. self-FMT). The error bars are standard deviations (*n =* 5). The limit of detection was 10^5^ GCE/g of stool. Ab, antibiotic; hFMT, human fecal microbiota transplantation; self-FMT, self-fecal microbiota transplantation.

We reasoned that in the intestinal niche of infants where RV develop, the resident microbiota determines infectivity. Then, we asked whether infection of RVwa (G1P[8]) in the mouse model could be improved by replacing its intestinal microbiota with the microbiota of infants by FMT using antibiotic (Ab)-mediated microbiota ablation. In order to answer this, C57BL/6J mice were treated with an antibiotic cocktail through drinking water for three weeks. Bacterial counts in fecal pellets decreased from 6.3–9.7 × 10^10^ CFU/g to no detectable counts (plating 10^−1^ dilutions), whereas they remained stable in the non-treated control (4.9 × 10^10^ CFU/g), indicating an efficient depletion of the culturable gut bacteria. Mice were then subject to FMT and orally dosed with the Wa strain. The virus could be detected in the feces of control animals with a peak at one day post-infection (dpi; 9.1 × 10^9^ genome copy equivalent (GCE)/g feces), which rapidly dropped, with RV being under the detection limit at dpi 4 (Figure 1). By the contrary, compared to control animals, at dpi 1 viral shedding in Ab-treated mice and in mice subject to human FMT (hFMT) was 23- and 16-fold higher (2.1 × 10^11^ GCE/g and 1.5 × 10^11^ GCE/g, respectively) and remained until dpi 5–6, following the same evolution pattern and with values higher than 10^9^ GCE/g. The lower levels of RV detected in control animals and its faster clearance were indicative of an inefficient replication, in contrast to the prolonged viral shedding in Ab-treated mice and in the hFMT group. In mice receiving a self-FMT, partial restoration of the restrictive replication of RVwa was evidenced, and virus shedding was detected at levels similar to control at dpi 1, with no detectable viral shedding at dpi 4 (Figure 1).

### 3.2. Impact of Fecal Microbiota Transplant on Mouse Microbiota

**Figure 2:**
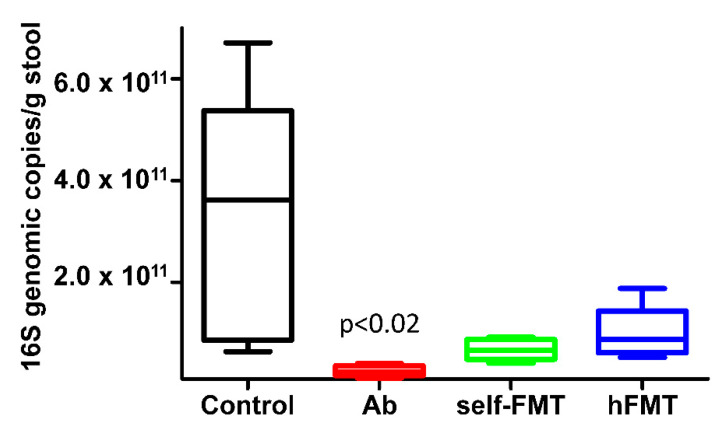
Total bacteria 16S DNA quantification by qPCR. The 16S DNA genomic copies/g of stool are presented for control mice, Ab-treated mice and mice subject to self-FMT and hFMT, respectively. Ab-treated mice had significantly lower amount of genome copies than the other three groups (*p <* 0.02). No significant differences were found between the human and self-transplanted groups.

The results of viral shedding pointed to microbiota changes by Ab treatment and hFMT as responsible for RVwa infection permissiveness in mice. The treatment with antibiotics significantly reduced the number of bacteria calculated by 16S rDNA qPCR (Figure 2). Both the transplant from human feces and the self-transplant increased the number of bacteria with no significant differences between them (Figure 2).

**Figure 3:**
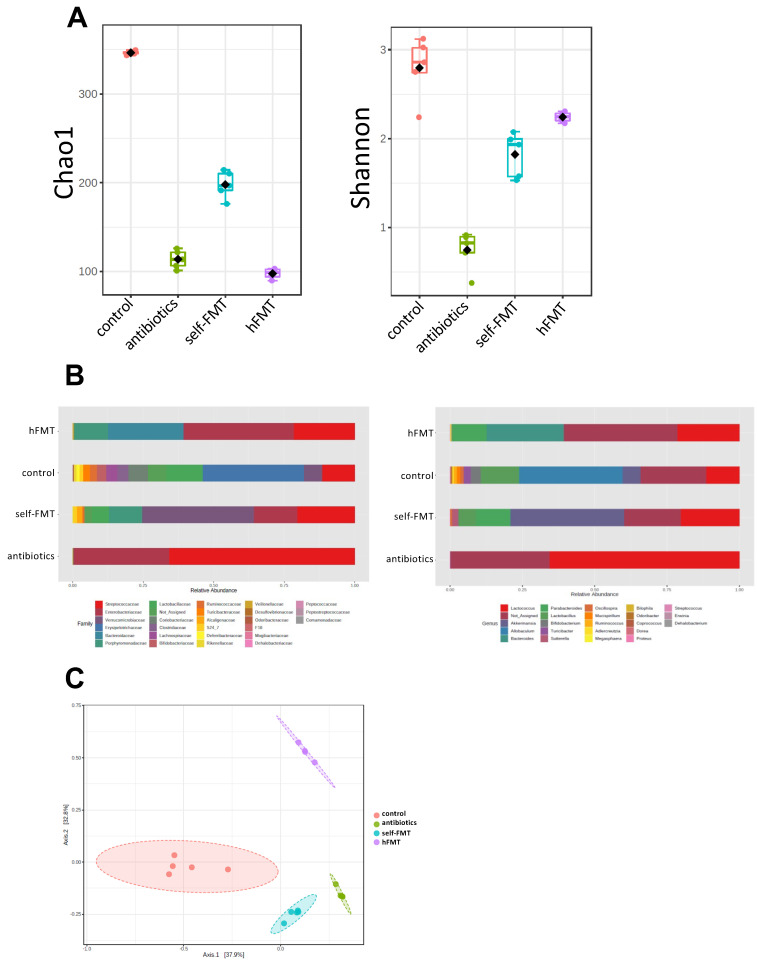
Changes in intestinal bacterial composition in the mice groups. (**A**) Microbial α-diversity. Microbial richness (Chao1) and diversity (Shannon) indexes are shown. (**B**) Relative abundance of bacterial taxa (family and genus levels) found in the feces of the different mice groups. (**C**) Differences in microbial global composition after the different treatments. A principal coordinate analysis (PCoA) of the Bray-Curtis dissimilarity indexes of samples is shown (PERMANOVA F-value = 36.77; R^2^ = 0.8803; *p <* 0.001).

The microbiota composition in feces prior to RV challenge was determined by 16S rDNA NGS, showing that bacterial richness and diversity was severely affected after the different treatments (Figure 3A). Chao1 index was lowered in all groups compared to control (microbial richness; *p =* 2.0365 × 10^−12^; F = 206.49, ANOVA) but was higher in self-FMT animals. Diversity (Shannon index) was also lower (*p =* 1.8988 × 10^−8^; F = 57.299, ANOVA), but was higher in the hFMT group compared to self-FMT group, indicating uneven microbial distribution after treatments. In both types of analyses Ab treatment resulted in lower α diversity. As expected, the three distinct treatments caused profound remodeling of the intestinal microbial profile and all groups could be differentiated in terms of overall composition (Figure 3B,C). In mice treated with Ab, members of the phyla *Firmicutes* and *Proteobacteria*, belonging to the genus *Lactococcus* and *unidentified* γ-Proteobacteria (enterobacteria), respectively, accounted for most of the microbiota (Figure 3B).

**Figure 4:**
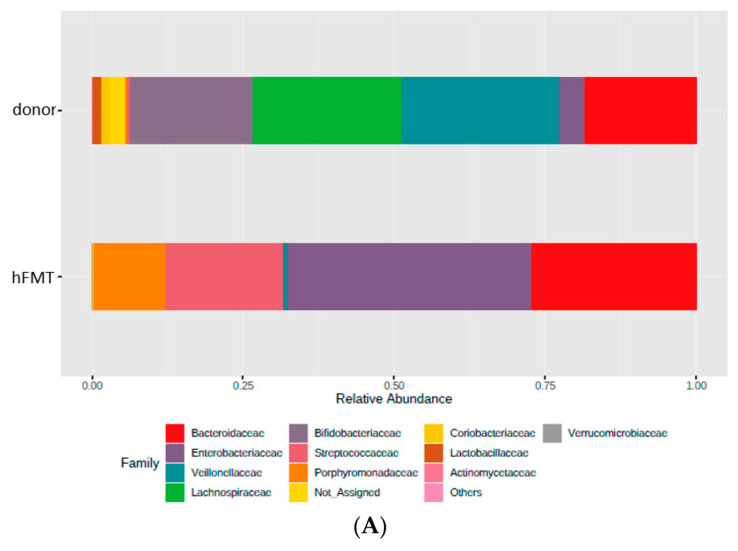

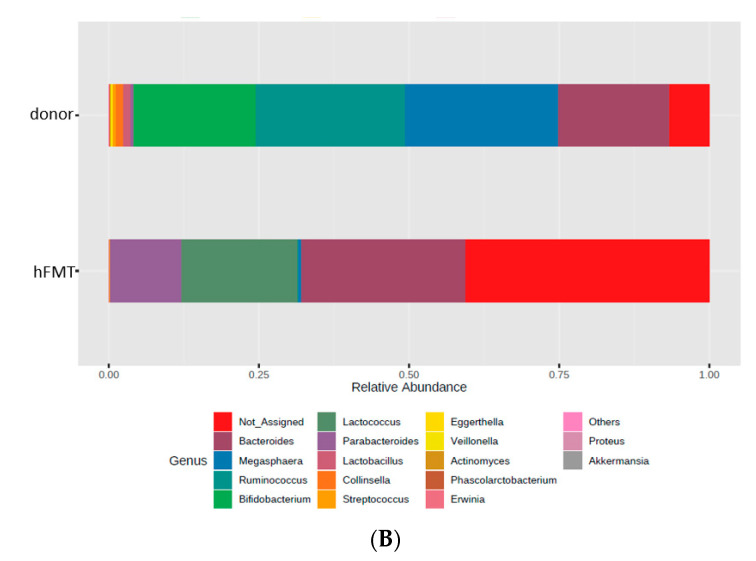
Relative abundances of bacterial taxa (family and genus) in donor feces and feces of mice subject to hFMT. The panel (**A**) shows the relative abundance of bacteria at family level of the donor sample while panel (**B**) shows the same at genus level.

**Figure 5:**
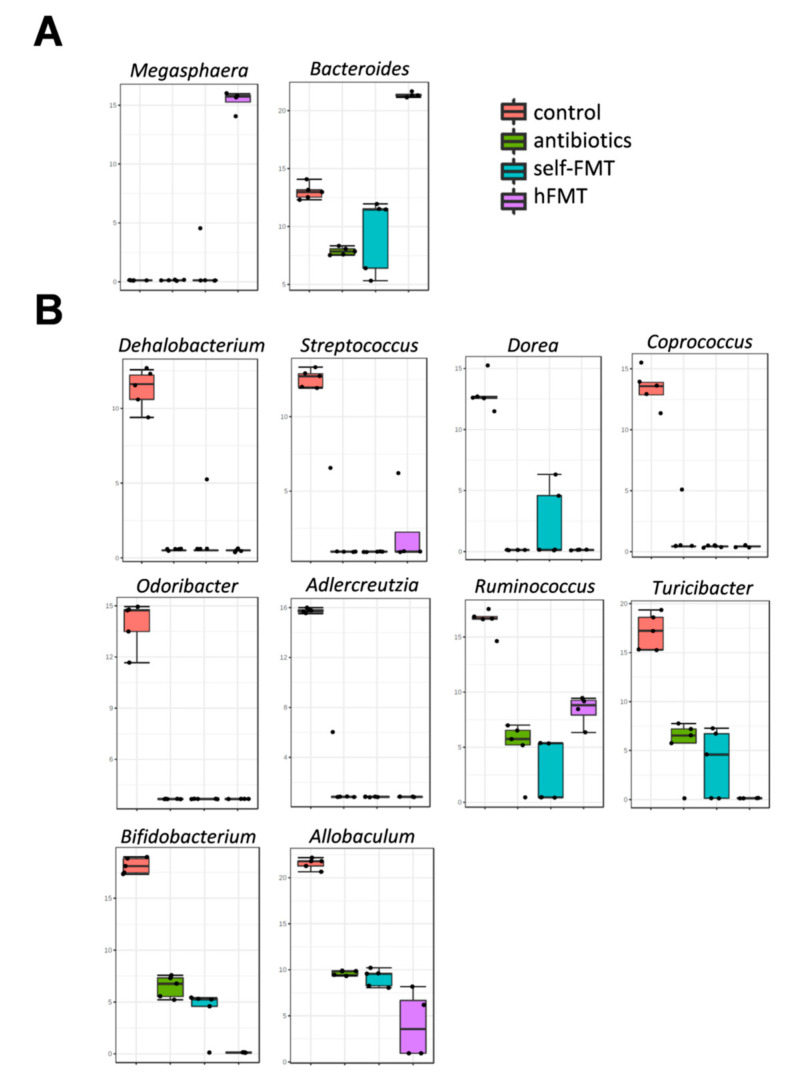

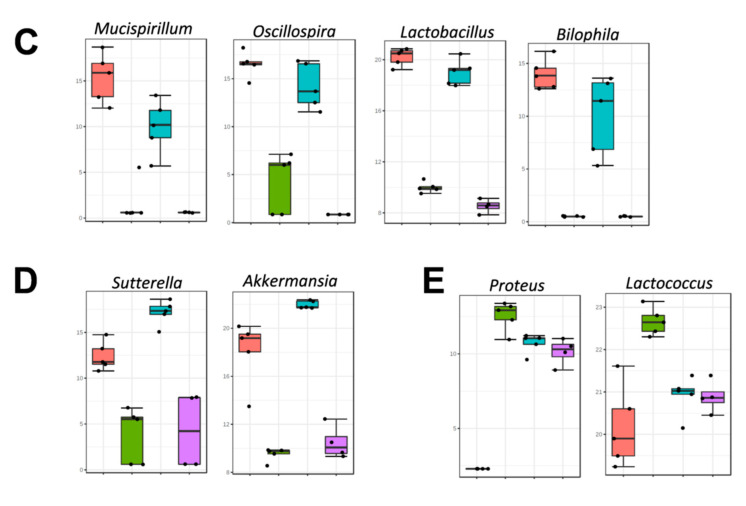
Bacterial genera showing differences between treatment groups. A LEfSe analysis was performed to detect bacterial markers characteristic of each treatment condition. The different box-plots represent abundance (log transformation of data normalized by total-sum scaling ×10^7^) of bacterial genera showing LDA scores >4. (**A**) Bacteria augmented after hFMT; (**B**) Genera ablated or diminished by Ab treatment; (**C**) Bacteria that were partially restored by self-FMT; (**D**) Bacteria augmented after self-FMT; (**E**). Bacteria increased in Ab-group. Red, control animals; green, antibiotics-treated animals; blue, self-FMT; magenta, hFMT.

The microbiota present in the pooled infant feces was not completely engrafted in mice after hFMT (Figure 4), but these mice were characterized by elevated numbers of Bacteroidetes and presence of members of the genus *Megasphaera*, a bacterial taxon that was absent in the microbiota of untreated mice (Figure 5A). Genera, such as *Bifidobacterium*, *Adlercreutzia*, *Ruminococcus*, *Coprococcus, Turicibacter*, *Odoribacter,* and *Allobaculum,* were completely depleted after Ab treatment and they were not replenished by any FMT, including self-FMT (Figure 5B). Although microbial engraftment was not completely successful in the self-FMT group (differences in α and β diversity were obvious compared to control animals), bacteria belonging to *Oscillospira*, *Lactobacillus*, *Mucispirillum,* and *Bilophila* were partially restored in mice via self-FMT (Figure 5C). In addition, in self-FMT animals proportions of *Akkermansia* and *Sutterella* taxons were increased compared to control mice (Figure 5D). In summary, low microbial richness and ablation of most representative taxa of mice microbiota was concomitant with higher mice permissiveness to RVwa infection.

### 3.3. Immune and Epithelial Glycosylation Gene Expression in the Gut Associates with Human Rotavirus Infection in Mice

**Figure 6:**
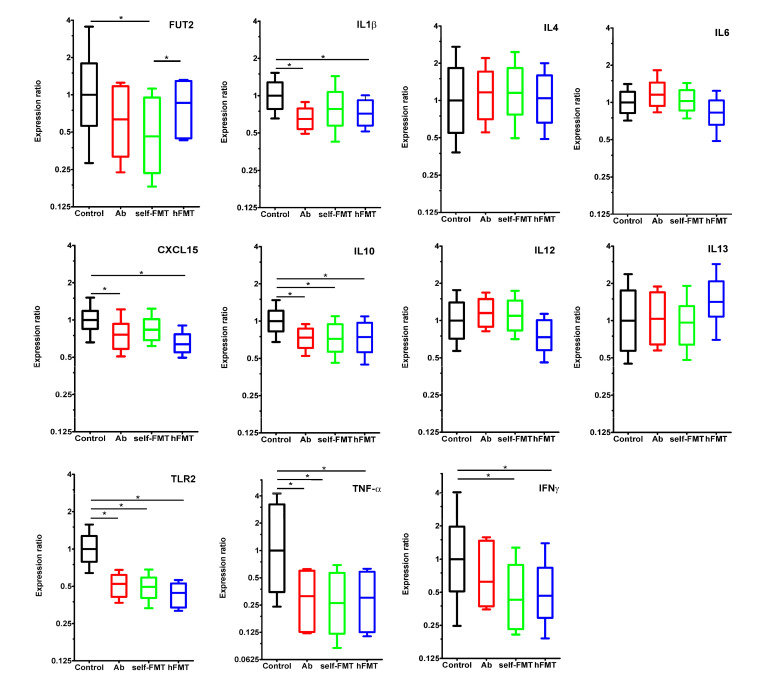
Differences in gene expression in the small intestine of infected mice. Expression of immune system mediators (*IL1β*, *IL4, IL6, CXCL15, IL10, IL12, IL13, IFNγ, TNFα,* and *TLR2*) and fucosyltransferase 2 (*FUT2*) was determined by RT-qPCR in mice from the different groups at 7 dpi (*n =* 5). The level of significance is indicated for control mice (* *p <* 0.05).

Gene expression levels of a panel of cytokines and other innate immune system mediators including *IL1β, IL4, IL6, CXCL15, IL10, IL12, IL13, IFNγ, TNFα,* and *TLR2* were studied by RT-qPCR in the small intestine of mice at 7 dpi (Figure 6). *IL6, IL12*, and *IL13* expression levels did not differ between experimental groups and control animals, whereas a downregulation effect was generally observed in the remaining tested genes. IL1β and CXCL15 messenger levels were lower in the Ab and hFMT groups where human rotavirus replicated more efficiently. Expression of *IL10, TLR2*, and *TNFα* was also reduced in all groups compared to control. Finally, *IFNγ* expression was diminished in mice subject to both FMT treatments. Expression of the *FUT2* gene, whose product is involved in α1,2-fucosylation during the synthesis of mucosal H type-1 antigen (fucosyl-α1,2-galactopyranosyl-β1,3-N-acetyl-glucopyranoside), one of the P[8] RV adhesin receptors, was downregulated in self-FMT animals. Interestingly, the *FUT2* gene expression differed between both transplanted groups, having a higher expression (*p =* 0.003) in the group that received the human feces transplant (Figure 6). More interestingly, a significant negative correlation was found between the expression level of *IL1β* and the rotavirus shedding in feces at 2 dpi (Pearson r = −0.7203, *p =* 0.0005). More moderate correlations were also found with *TLR2* (Pearson r = −0.5824, *p =* 0.0089) and *TNFα* (Pearson r = −0.4654, *p =* 0.0446).

## 4. Discussion

Gut microbiota has emerged as a pivotal player in enteric virus-host interactions, and has been shown or hypothesized to have positive, as well as negative effects on viral infectivity, mediated by different mechanisms [2,3,4,5]. Given the lack of an efficient mouse model to replicate relevant human RV strains [13], we studied whether mice could be infected with RVwa after an infant gut microbiota engraftment. This RV strain has been used as a model for human RV studies and has special relevance because it carries the P[8] VP4 genotype, which is responsible for around 90% of infections [22]. An available in vivo model of replication would therefore be desirable, yet its infectivity in small animal models is not satisfactory.

In our study, RVwa infection could not be linked to hFMT, ruling out the possibility that human intestinal microbiota mediates RVwa infectivity in mice. Nonetheless, we show that independently of subsequent hFMT, simple gut bacteria depletion through antibiotic treatment dramatically increased the replication capacity of the RVwa strain in this host. Altered intestinal bacterial loads in the mice do not account for the differences in RVwa replication, as no differences were found in bacterial numbers in the feces from groups that substantially differed in RVwa infection. It has been discussed that variations in the microbiome of experimental animals at different laboratories may affect mice phenotypes, which can be at the basis of reproducibility issues [23]. This situation would have minor effects in our case, as we showed that the sole fact of depleting the microbiota with antibiotics allowed RVwa replication. Our data suggest that endogenous mouse microbiota restricts RVwa replication, which is in opposition to results obtained with the murine RV EC strain, where Ab treatment had negative effects on viral entry, delaying and decreasing infectivity in both adult (40% reduction in viral shedding) and neonatal (34% reduction in diarrhea incidence) murine models [8]. However, recent research also points to a protective role for the microbiota during murine RV (strain EDIM) infection, by demonstrating high susceptibility to RV infection in Ab-treated and germ-free animals and highlighting a role of microbiota-induced *IL22* expression as an anti-viral mediator [24].

The importance of the microbiota in establishing an appropriate anti-viral immune response has been noted [25,26]. Exacerbated systemic infection by several enteric and non-enteric viruses was observed in Ab-treated mice, including vesicular stomatitis and influenza virus [27], murine gamma herpesvirus [28], respiratory syncytial virus [29], encephalomyocarditis virus [30], and West Nile, Dengue, and Zika viruses [31]. In some cases, microbiota depletion has been linked to a defective innate immune response characterized by low levels of type I IFN expression (IFNβ), which hampered the ability to mount an effective anti-viral macrophage response [27,29]. In our experiments, antibiotic treatment generally resulted in reduced expression of inflammatory mediators in the small intestine, although no *IFNβ* expression could be detected in the whole tissue (data not shown). We found that *IL10* and *TNFα* displayed reduced expression in microbiota-depleted animals and that *IFNγ* expression was also lower in FMT animals. These are important mediators of immune response to infections, including RV, in which studies have previously reported increased expression after infection in a mouse model [32,33]. Accordingly, although germ-free mice show delayed infection with EC RV, viral clearance in this model was found to last substantially longer, probably reflecting an immature intestinal immune system [8]. *IL1β* expression was lowered in Ab and hFMT groups and negatively correlated with viral shedding. This cytokine plays a major role in early inflammatory response against pathogens and its level is increased during RV infection in children [34]. Therefore, reduced IL10, IL1β, TNFα, and IFNγ are likely to be beneficial for viral replication. *TLR2*, whose expression was lowered in the Ab-treated groups, has been linked to the protective effect of lactobacilli towards RV infection in animal models [35,36]. We also studied the association between RV infection and FUT2. It was previously shown that specific members of the gut microbiota, such as *Bacteroides thetaiotaomicron*, *Bacteroides fragilis,* and segmented filamentous bacteria (SFB) are able to influence the pattern of mucosal glycosylation by inducing epithelial *FUT2* expression [10,37], which encodes the enzyme responsible for H-type antigen synthesis, the target for binding of RV P[8] adhesin [11]. We showed that *FUT2* expression in the small intestine was only reduced in self-FMT animals. Lack of intestinal microbiota generally results in downregulation of *FUT2* [37], and it is not known how diminished *FUT2* levels could affect RVwa infection in mice, especially given that another entry mediator of P[8] RV, the Lewis b antigen [12], is not produced in this host. A recent report shows that virulent RVwa (a strain serially passed in gnotobiotic pigs) infection in porcine enteroids is enhanced by the presence of H antigen [38], but the role of HBGA in infection in mice has yet to be determined.

The limited RVwa infection found in self-FMT animals suggests that specific microbial taxa not implanted after hFMT are implicated in RV exclusion. Although self-FMT did not restore the original microbiota of mice, several bacterial taxa with a potential role in RVwa restriction were partially restored: *Mucispirillum* is a strict anaerobe typically found in the murine intestinal tract, where it is intimately linked to the mucosal layer and participates in inflammatory processes [39] and is also involved in excluding several pathogenic bacteria [40]; *Oscillospira* is another anaerobe from the grastrointestinal tract, and, although it has never been cultivated, evidence indicates its relevance to human health [41]; *Bilophila*, which comprises the single species *Biliophila wadsworthia*, has been linked to intestinal inflammation in mice [42]; and finally, lactobacilli are well-known probiotic bacteria that have been shown to diminish RV-induced diarrhea in humans [43] and reduce RV infection in both in vitro and in vivo models [44,45].

A recent study showed that members of the murine intestinal microbiota such as SFB belonging to *Candidatus* Arthromitus are responsible for inhibiting murine RV EC strain infection [46]. Thus, Ab elimination of SFB could explain Ab-treated mice tolerance to RVwa infection. However, SFB were not found in our microbiota analysis and the model where they were implicated in resistance to the murine RV strain EC used immunosuppressed Rag1-KO mice, which are defective in B and T lymphocytes. However, *Candidatus Arthromitus* was not able to stably colonize normal mice [46]. Nevertheless, the idea that some bacterial taxa, such as those re-established after self-FMT or others, could mediate RVwa exclusion in mice is still attractive. Such a category of bacteria has been identified and the anti-viral mechanisms behind them disclosed in some cases. As examples, oral dosing of *Blautia coccoides* in Ab-mice restored the capacity of macrophages to induce IFNβ and promoted protection against encephalomyocarditis virus systemic infection [30], and, likewise, lipo-oligosaccharides from the outer membrane of Bacteroides and microbial metabolism-derived acetate were involved in triggering an IFNβ response that prevented vesicular stomatitis virus and influenza virus [27] and respiratory syncytial virus [29] infection in mice, respectively.

RVwa permissiveness in Ab-treated mice could also derive from depletion of one or various of the many microbial taxa affected by this treatment but not restored by self-FMT. As an example, *Ruminococcus* practically disappeared after Ab administration. These bacteria were correlated with low anti-RV IgA titers in human saliva of healthy subjects [47], have recently been characterized as microorganisms physically interacting with RV in the stools of children suffering P[8] RV-induced diarrhea, and reduced RVwa infectivity in Caco-2 cell cultures [9]. *Bifidobacterium*, another relevant genus of probiotic bacteria with proven anti-RV properties [45,48] was also eradicated by antibiotics.

Alternative mechanisms by which bacteria could mediate RV protection include direct interaction of RV virions with bacteria, as hypothesized for SFB [46]. These interactions have been evidenced in in vitro experiments for a number of bacterial strains [32,49]. Species such as *Ruminococcus gauvreauii* [9] and others [50] express HBGA-like substances on their surface which are binding targets for RV. HBGAs act as virion stabilizers that promote bacteria-assisted binding and enhance infection in some enteric viruses, such as norovirus (NoV) and poliovirus [51,52,53] but can mediate virus sequestration on the bacterial surface, which could prevent virus from binding to target cells.

The mechanisms underlying RVwa development in antibiotic-treated mice are as yet unknown, but our results suggest either that natural mouse resistance to RVwa infection is triggered by indigenous mouse commensals that cannot be substituted by other human intestinal bacteria, or, alternatively, that the equivalent human microorganisms cannot be implanted in the mouse by simple FMT. Further characterization of the factors mediating RV replication in Ab-treated mice is needed. Nonetheless, this in vivo infection model may constitute a valuable tool to investigate the biology of RV. Identifying the bacteria responsible for human RV restriction in mice will further understanding of the relationship established between RV and intestinal commensals.

## Data Availability

The raw sequencing fastq files were deposited in the SRA repository (http://www.ncbi.nlm.nih.gov/sra accessed 1 July 2021) under Bioproject ID PRJNA706108.

## Supplementary Materials

The following are available online at https://www.mdpi.com/article/10.3390/biomedicines9070846/s1, Table S1: primers used for qPCR in the present study.
