# Supplementary material for: Microbiota Depletion Promotes Human Rotavirus Replication in an Adult Mouse Model
Source: Biomedicines. 2021 Jul 20;9(7):846. doi: 10.3390/biomedicines9070846 (PMC8301474; doi:10.3390/biomedicines9070846)
Supplement: Supplementary file 1 [file biomedicines-09-00846-s001.zip › biomedicines-1290884-supplementary.pdf]

biomedicines

MDPI

Article

Microbiota Depletion Promotes Human Rotavirus Replication in an Adult Mouse Model

Roberto Gozalbo-Rovira [1,†], Cristina Santiso-Bellón [1,†], Javier Buesa [1,2], Antonio Rubio-del-Campo [3], Susana Vila-Vicent [1], Carlos Muñoz [1], María J Yebra [3], Vicente Monedero [3,*] and Jesús Rodríguez-Díaz [1,2,*]

1 Department of Microbiology, School of Medicine, University of Valencia, Av. Blasco Ibáñez 17, 46010 Valencia, Spain; rovigoro@uv.es (R.G.-R.); cristina.santiso@uv.es (C.S.-B.); javier.buesa@uv.es (J.B.); susana.vila@uv.es (S.V.-V.); carlos.munoz@uv.es (C.M.)
2 Hospital Clínico Universitario de Valencia, Instituto de Investigación INCLIVA, Valencia, Spain
3 Department of Biotechnology, IATA-CSIC, Av. Agustín Escardino 7, 46980 Paterna, Valencia, Spain; anrucam@iata.csic.es (A.R.d.C.); yebra@iata.csic.es (M.J.Y.)
* Correspondence: btcmon@iata.csic.es (V.M.); jesus.rodriguez@uv.es (J.R.-D.); Tel.: +34-96-3983316 (V.M.); +34-96-3900022 (ext. 2006) (J.R.-D.)
† These two authors contributed equally.

Table S1. primers used for qPCR in the present study.

Target	Gene Bank Access number	Primer name	Sequence	Nucleotide positions	Reference
IL1β	NM_008361	IL-1β-F	5′-TCGCTCAGGGTCACAAGAAA-3′	995-1014	[1]
		IL-1β-R	5′-CATCAGAGGCAAGGAGGAAAAC-3′	1067-1046	
IL4	NM_021283.2	IL-4-F	5′-GGTCTCAACCCCCAGCTAGT-3′	63-82	[2]
		IL-4-R	5′-GCCGATGATCTCTCTCAAGTGAT-3′	164-142	
IL6	NM_031168	IL-6-F	5′-CTGCAAGAGACTTCCATCCAGTT-3′	92-114	[1]
		IL-6-R	5′-GAAGTAGGGAAGGCCGTGG-3′	161-143	
CXCL15	NM_011339	IL-8-F	5′-CAGGCCACAGACGGACATG-3′	22-40	[1]
		IL-8-R	5′-GGACGAAGATGCCTAGGTTAAGG-3′	98-76	
IL10	NM_010548	IL-10-F	5′-GCTCTTACTGACTGGCATGAG-3′	97-117	[1]
		IL-10-R	5′-CGCAGCTCTAGGAGCATGTG-3′	201-182	
IL12 (IL12p40)	AH004859.1	IL-12-F	5′-AGCACCAGCTTCTTCATCAGG-3′	720-740	[3]
		IL-12-R	5′-CCTTTCTGGTTACACCCCTCC-3′	931-911	
IL13	NM_008355.3	IL-13-F	5′-CCTGGCTCTTGCTTGCCTT-3′	92-110	[2]
		IL-13-R	5′-GGTCTTGTGTGATGTTGCTCA-3′	207-187	
TNFα	NM_013693	TNF-α –F	5′-CCACCACGCTCTTCTGTCTAC-3′	298-318	[1]
		TNF-α-R	5′-TGGGCTACAGGCTTGTCACT-3′	448-429	
INFγ	NM_008337.4	INF-γ-F	5′-CATTCATGAGTATTGCCAAGTTTG-3′	444-467	[4]
		INF-γ-R	5′-GCTGGATTCCGGCAACAG-3′	547-530	
TLR2	NM_011905.3	TLR2-F	5′- GTCTCTGCGACCTAGAAGTGGA -3′	162-183	[5]
		TLR2-R	5′-CGGAGGGAATAGAGGTGAAAG A-3′	497-476	
GAPDH	NM_001289726	GAPDH-F	5′-AGCTTGTCATCAACGGGAAG-3′	283-302	[1]
		GAPDH-R	5′-TTTGATGTTAGTGGGGTCTCG-3′	344-324	
RPLP0	NM_007475.5	RPLP0-F	5′-AGATTCGGGATATGCTGTTGGC-3′	411-432	In house

References.

1. Leclercq S, Mian FM, Stanisz AM, Bindels LB, Cambier E, Ben-Amram H, et al. Low-dose penicillin in early life induces long-term changes in murine gut microbiota, brain cytokines and behavior. Nat Commun. 2017;8: 15062. doi:10.1038/ncomms15062

2. Spandidos A, Wang X, Wang H, Seed B. PrimerBank: a resource of human and mouse PCR primer pairs for gene expression detection and quantification. Nucleic Acids Res. 2010;38: D792-9. doi:10.1093/nar/gkp1005
3. Cardona P-J, Gordillo S, Diaz J, Tapia G, Amat I, Pallares A, et al. Widespread Bronchogenic Dissemination Makes DBA/2 Mice More Susceptible than C57BL/6 Mice to Experimental Aerosol Infection with Mycobacterium tuberculosis. Infect Immun. 2003;71: 5845–5854. doi:10.1128/IAI.71.10.5845-5854.2003
4. Keilbaugh SA, Shin ME, Banchereau RF, McVay LD, Boyko N, Artis D, et al. Activation of RegIIIbeta/gamma and interferon gamma expression in the intestinal tract of SCID mice: an innate response to bacterial colonisation of the gut. Gut. 2005;54: 623–9. doi:10.1136/gut.2004.056028
5. An H, Yu Y, Zhang M, Xu H, Qi R, Yan X, et al. Involvement of ERK, p38 and NF-kappaB signal transduction in regulation of TLR2, TLR4 and TLR9 gene expression induced by lipopolysaccharide in mouse dendritic cells. Immunology. 2002;106: 38–45. doi:10.1046/j.1365-2567.2002.01401.x
